# Community Participatory Co-Design and Development of a Digital Diabetes Prevention Education Program for Hispanic Families With Obesity: Mixed Methods Study

**DOI:** 10.2196/67800

**Published:** 2026-02-11

**Authors:** Sandra Mihail, Marbelly Partida, Lizette Villanueva, Debbe Thompson, Teresia M O'Connor, Salma M Musaad, Maria J Redondo, Erica G Soltero

**Affiliations:** 1 USDA/ARS Children's Nutrition Research Center, Department of Pediatrics, Baylor College of Medicine, 1100 Bates St., Houston, TX, 77030, United States, 1 713-798-7154; 2 Division of Pediatric Diabetes and Endocrinology, Department of Pediatrics, Baylor College of Medicine, Houston, TX, United States

**Keywords:** digital health, Hispanic health, diabetes prevention, family-based intervention, digital education, obesity, digital health intervention, disease prevention, diabetes, DM, diabetes mellitus, type 2 diabetes, type 1 diabetes, endocrinology, adolescents, digital devices, digital platform, feasibility pilot study, evidence-based interventions

## Abstract

**Background:**

Digital health interventions (DHIs) can extend the reach of disease prevention interventions; however, few are evidence-based, theoretically grounded, or developed for high-risk youth and families. Co-design approaches engage end users in the design and development of the DHI, which can lead to increased accessibility and engagement.

**Objective:**

This study aimed to describe the adaptation of an evidence-based diabetes prevention program for remote, digital delivery.

**Methods:**

The adaptation of the in-person intervention was guided by a modified Inclusive Digital Health Intervention Design to Promote Health Equity framework and conducted in collaboration with Hispanic adolescents (n=23) with obesity (BMI ≥95th percentile) and their parents (n=15). Focus groups identified digital, health education, and support needs. An expert and community panel assisted in developing solutions based on these findings. A sample content session with a food tasting experience was created and reviewed by participants. The research team subsequently built a digital platform to host the content. Participants assessed the usability of the platform, including the ease of use, design components, and technical issues. A second meeting of the expert panel provided recommendations for further refinement and feedback.

**Results:**

Findings from focus groups indicated that most participants (31/36, 86.1%) reported stable internet access and multiple digital devices. With regard to format, a few parents (2/9, 22.2%) preferred synchronous content sessions, while most youth and parents favored asynchronous sessions (7/9, 77.8%) lasting 40 to 60 minutes. Health education needs included interactive content, healthy recipes, and the ability to ask questions. Experts suggested offering asynchronous sessions with monthly synchronous meetings to meet both parent and youth needs. After viewing a sample session, families found the content easy to understand and mostly engaging, with (17/21) 81% participating in the food tasting activity and all participants reporting that the activity was feasible. Experts recommended using a more conversational, interactive teaching style to improve the content and using a food box with nonperishable items to increase the ease of food tasting activities. While the digital platform was functional and easy to use, families highlighted the need for larger font and icon sizes, easier navigation, and better color contrasts. On the basis of this feedback, experts advised creating tutorial videos and an orientation session for platform training. The content and platform will continue to be refined before further evaluation in a 12-week feasibility pilot study.

**Conclusions:**

The use of a co-design approach provided opportunities to make content more interactive and engaging and to increase the ease of use of the digital platform. Describing the adaptation process using a guiding framework in collaboration with the focus population will inform future studies aiming to adapt evidence-based interventions to a digital platform.

## Introduction

The rapid advancement of digital technology in the health sector has led to developments promising enhanced interventions, increased patient engagement, and improved outcomes [1]. Digital health interventions (DHIs) that are thoughtfully designed, equitably implemented, and effectively used have the potential to address health disparities and build equitable disease prevention solutions. For example, Hispanic youth are disproportionately affected by obesity and consequently are the most insulin-resistant pediatric subgroup in the United States [2,3]. DHIs are especially promising for today’s youth, who are digital forerunners with more exposure to and use of digital technology compared to previous generations [4]. Smartphone ownership among Hispanic youth is comparable to that of non-Hispanic White youth (95% vs 94%); adolescents are the highest users of SMS text messaging, averaging 60 messages a day; and nearly two-thirds of adolescents report using a smartphone app to support changes in diet or physical activity [5]. These digital tools are familiar, relevant, and potentially cost-effective, given the high rate at which they are currently owned and used by youth across sociodemographic levels. However, there are many digital tools and services in the marketplace that are not developed with the guidance of health behavior change experts, nor are they evidence-based, limiting their ability to lead to clinically meaningful changes in disease outcomes [6-8].

In addition to the need for more evidence-based DHIs, there is also a need for DHIs with equitable design and access, especially among high-risk populations [7]. Apart from access to digital tools and services, engagement in DHIs also requires a high level of digital literacy and self-efficacy [9,10]. For DHIs to be effective, individual- and environment-level digital determinants must be considered, such as design needs, user preferences, and community infrastructure available among the focus population [11,12]. One strategy for establishing equitable access and identifying the individual and environmental determinants that impact engagement and participation in DHIs is to collaborate with the end user in the design and development process. Using community-engaged strategies to foster collaboration with the end user can assist in the development of DHIs that integrate the end user’s background and contextual determinants into the design and implementation of the program [13]. Without equitable design and access, digital health strategies can potentially contribute to and exacerbate health and digital disparities [9,10]. Unfortunately, few studies have engaged youth in the design and development process, and few DHIs have been developed and tested among Hispanic youth [9,10].

To address current limitations in DHIs and digital equity, there is a need for more evidence-based solutions that are co-designed with the focus population. To date, only one culturally grounded, family-based type 2 diabetes (T2D) prevention program has been developed and rigorously tested among Hispanic adolescents and families [14-16]. This study was developed using core constructs of the national diabetes prevention program and has demonstrated feasibility, acceptability, and efficacy for improving insulin sensitivity, adiposity, and delaying the onset of T2D when implemented in a community setting [15,17]. The purpose of this study was to adapt an evidence-based diabetes prevention program for remote, digital delivery among Hispanic adolescents with obesity and their families. The objective of this manuscript was to describe the use of community-engaged strategies to guide the adaptation process to ensure that the adapted DHI is co-designed with Hispanic youth and meets the health and digital needs of high-risk Hispanic youth and families. By detailing the adaptation process, this manuscript will inform future studies aimed at adapting evidence-based disease prevention programs in collaboration with the focus population for delivery via digital tools and services.

## Methods

### Participants

Participants were recruited from the greater Houston metropolitan area through collaborations with local pediatric clinics (5/36, 13.8%), the United States Department of Agriculture Children’s Nutrition Research Center volunteer research database (3/36, 8.3%), community organizations (21/36, 58.3%), and word of mouth (7/36, 19.4%). Interested participants were screened for inclusion and exclusion criteria via telephone. Participants met the inclusion criteria if they self-identified as Hispanic or Latino, had obesity (BMI ≥95th percentile and <120% of the 95th), were between the ages of 14 and 16 years, and owned a mobile smartphone. Participants were excluded if they had a diagnosis of T2D, were taking medication, or had received a previous diagnosis of a condition that influences activity, sleep, or cognition. Families were informed that this study included a series of 3 community-engaged activities. All participants were invited to all 3 community-engaged activities; however, families were also informed that participation was voluntary and that they could participate in as many or as few activities as they desired.

### Ethical Considerations

The study protocol and all study-related materials were approved by the Institutional Review Board at Baylor College of Medicine (H-50331). Before any study procedures, trained research team members obtained written parental consent and child assent. All study-related documents were available in English and Spanish, with bilingual or bicultural research staff administering consent, conducting data collection, and answering questions. Participants were informed that their participation was voluntary, and they were free to withdraw from the study at any time. Participants were also informed that nonparticipation would not affect any health or medical services they currently receive and that confidentiality would be maintained using unique identification numbers and a password-protected database. This study is registered at ClinicalTrials.gov (identifier: NCT06943001). Participants were compensated US $25 for participation in virtual focus groups and surveys and US $30 for the in-person think-aloud assessment.

### Evidence-Based Intervention

The evidence-based intervention to be adapted in this study draws from the national Diabetes Prevention Program (DPP). Using a rigorous process, core constructs from the DPP were culturally grounded and tailored for Hispanic youth and families in collaboration with community stakeholders [18]. The resulting nutrition and well-being curriculum includes 12 family-based, group sessions (Table 1). Sessions are adapted to meet the developmental needs of adolescents and to integrate Hispanic cultural values such as *respeto* (respect) and *familismo* (familism). For example, the value and importance of family are emphasized throughout the curriculum, and family-focused “homework” assignments are included, such as preparing a healthy meal together or going on a walk as a family [16]. Traditionally, the intervention is delivered in person by bilingual or bicultural implementers in 1-hour sessions, once a week. The intervention promotes changes in diet and physical activity by fostering social support and enhancing self-efficacy within families, between participating families, and among families and the implementers. Every session begins with a tasting experience in which youth and families are exposed to a healthy snack and are encouraged to engage in mindful eating practices. Every session has 30 to 40 minutes of nutrition education content with clear learning objectives, and the content builds continuously from week to week. At the end of each session, families are encouraged to set a behavior change goal that is specific, measurable, achievable, relevant, and time-bound (SMART). Time is also allotted for families to discuss and share information on successes and challenges with their behavior change goals [10,16,17].

**Table 1.** Curriculum topics.

Topic number	Topic
1	Getting started
2	Health awareness
3	Roles and responsibilities
4	Keep it moving
5	How sweet are you?
6	Champions with breakfast
7	Slim the fat
8	Fast food
9	Snack attack
10	Stay strong
11	Self-esteem
12	Balancing act

### Adaptation Framework

#### Co-Design Framework

**Figure 1.**
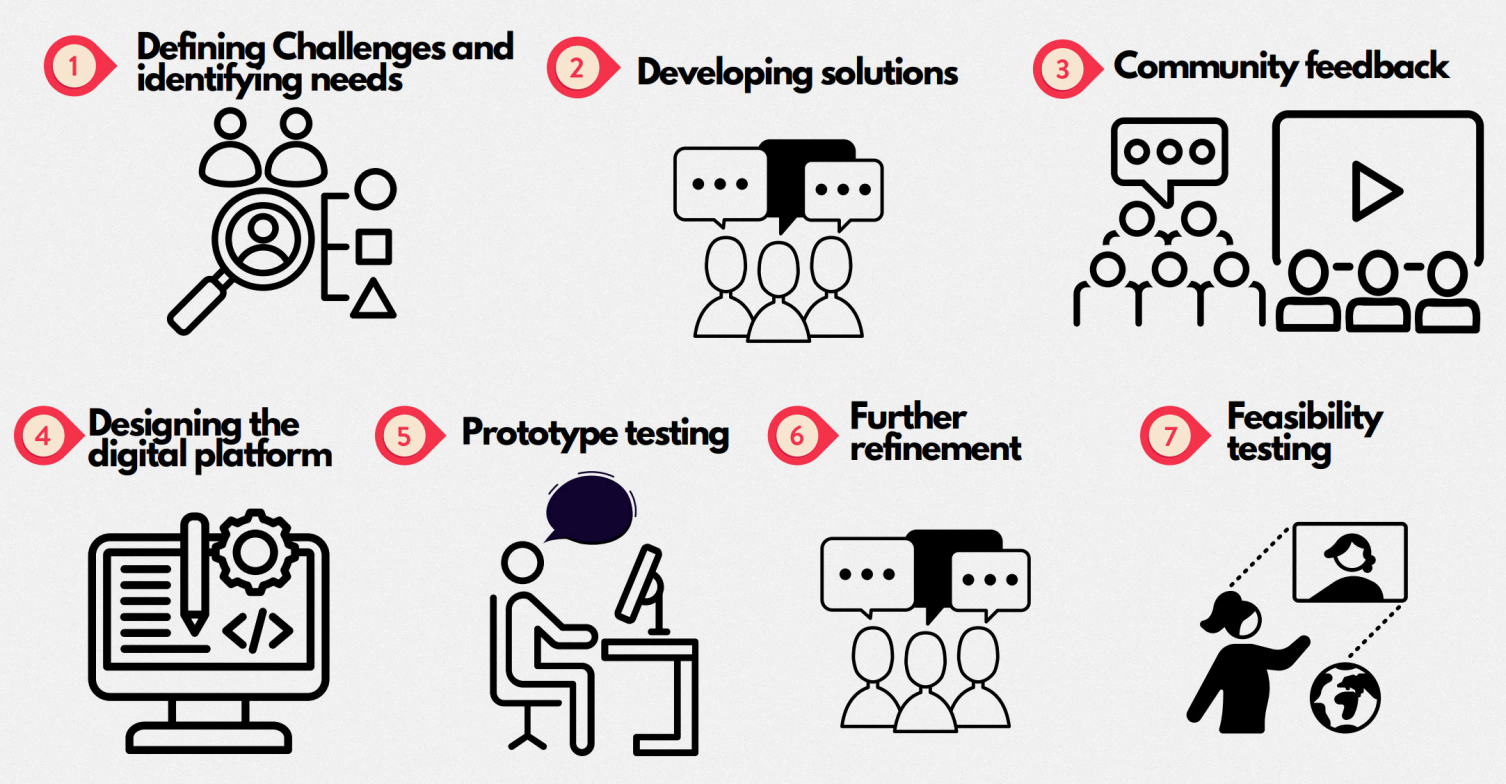
Modified inclusive digital health intervention design to promote health equity framework.

To adapt the in-person intervention for delivery via a remote, digital platform, we used a modified version of the Inclusive Digital Health Intervention Design to Promote Health Equity or iDesign framework (Figure 1). This framework was selected as it centers on the needs, values, and preferences of the end user. iDesign provides a structured framework for guiding the design of DHIs by applying community-engaged principles, including the end user, and addressing challenges in engaging diverse populations [19]. To fulfill the objective of this study, we used the 7 steps within the iDesign framework to guide the adaptation process. The study activities performed in each step are described in the following sections.

#### Step 1: Defining Challenges and Identifying Needs

In step 1, youth (n=12) and parents (n=9) were invited to participate in separate focus groups (n=5 per group), which took place via a private, secure Zoom link (Zoom Communications, Inc) or at a convenient community location from November 2022 to January 2023. Focus groups were audio recorded to capture community input. Following the focus groups, youth (n=15) were invited to participate in an interactive survey. This survey included multiple-choice and short-answer questions. Using an audio recording function, participants could respond to short-answer questions to provide narrative responses. This was done to obtain in-depth information while removing the burden of writing out their answers. The goal of the focus groups and the interactive survey was to elicit information on the (1) preferred format and timing of intervention sessions, (2) diet and physical activity education needs, (3) technology within the home environment, (4) guidance on adapting in-person activities to a digital platform, and (5) strategies for making the intervention engaging and fun for youth. Data collected from step 1 were analyzed and used to develop a report.

#### Step 2: Developing Solutions

In step 2, a 1.5-hour workshop-style meeting was conducted with a panel of experts (n=5) in May 2023. Experts consisted of medical and research professionals with experience and expertise in pediatrics, endocrinology, community-engaged research, diabetes prevention, and digital health. The data report from step 1 was presented to the expert panel. The goal of this meeting was to obtain feedback on data from step 1 and to brainstorm solutions for key needs and barriers expressed by participants, including (1) the format of the digital intervention, (2) family engagement and interaction, and (3) strategies for meeting the health education needs of families. Feedback on these key areas was added to the data report and used to develop a preliminary plan for adapting the evidence-based intervention. The preliminary adaptation plan was sent to 2 community health experts who led the development of and currently implement the original curriculum in community and clinic settings. The community health experts were asked to ensure that key components within the intervention were retained while integrating new adaptations to meet family preferences and needs expressed in the focus group.

#### Step 3: Community Feedback

In step 3, feedback from research and community experts was used to develop and record a sample nutrition education and wellness session. Most of the families (n=16) were invited to watch the sample session from their home environment and were asked to complete an evaluation survey following their viewing (Multimedia Appendix 1). Two families (n=5) were asked to attend a live viewing of the sample session via Zoom, with the research team in attendance. Following the live viewing, a focus group was conducted among participants to elicit more in-depth feedback on the session content, the level of engagement of the content, the evaluation of the teaching style, and perceptions of the length of the session and to elicit suggestions for improvement. This focus group was audio recorded to fully capture feedback on the sample session. Families that attended the live viewing were also asked to complete an evaluation survey following their viewing.

#### Step 4: Designing the Digital Platform

In step 4, the research team focused on developing an online learning platform that could meet the needs that were discussed in step 1. The initial features considered in the design of the digital online platform included hosting video content, the ability to upload additional resources such as PDFs, a messaging system to ask questions regarding educational content and report technical issues, and a platform that could be password protected to maintain participant confidentiality. On the basis of counsel from the expert panels, we focused on a platform that could measure metrics associated with participant engagement (eg, interactions with content, logins, and activities completed).

#### Step 5: Prototype Testing

In step 5, youth and families from step 1 were invited to participate in a “think-aloud” assessment using a prototype of the platform. Four adults and 4 teens participated in this assessment. Parents were asked to complete the assessment on a laptop computer, while youth were asked to complete the assessment on their own mobile smartphone to test the usability on both types of devices. Participants were given 15 minutes to complete six tasks: (1) navigate to the website, (2) create an account with a username and password, (3) watch a sample from lesson one (2 min), (4) complete a brief activity for lesson 1, (5) post their activity answers on the discussion board, and (6) use the “Ask the Instructor” feature to ask a nutrition-related question from the video. Using the “think-aloud” approach, participants were asked to voice their thoughts while performing the 6 tasks. Research team members observed participants while they completed the assessment and rated the degree to which participants completed every task and noted comments made by participants. Immediately following the assessment, parents and teens were engaged in a follow-up focus group to elicit feedback on how to improve the design, ease of use, and functionality of the platform. Research team members also asked questions about tasks that seemed difficult to complete, comments that were made during the assessment that needed clarification, and other observations made during the assessment.

#### Step 6: Further Refinement

In step 6, another 1.5-hour workshop-style meeting was conducted via Zoom with our panel of research experts in May 2024. The purpose of this meeting was to review findings from the “think-aloud” assessment and allow the expert panel to make additional recommendations for improving the design and usability of the digital platform.

#### Step 7: Feasibility Testing (Future)

In preparation for this step, we will prepare for the implementation of a 12-week feasibility pilot study by incorporating all the feedback gained throughout the previous steps into the development of the digital diabetes program. This includes modifying the learning platform and filming and recording all nutrition education and wellness sessions in English and Spanish. Feasibility testing will be conducted in a larger group of patients (n=48).

## Results

### Step 1 Results

Audio from focus groups was transcribed using a medical professional transcription company, and data gathered from focus groups and interactive surveys were coded by 2 trained, independent coders and were qualitatively analyzed using NVivo version 9 (QSR International). Thematic content analysis was used to identify emergent patterns and insights from focus groups and interactive surveys. Findings from the focus groups and interactive surveys revealed the desired format of the digital intervention, health education, and technology needs of Hispanic youth and families. With regard to the format of intervention sessions, most participants (27/36, 75%) preferred virtual, asynchronous sessions compared to hybrid (asynchronous or synchronous; 5/36, 13.9%) or synchronous (4/36, 11.1%) sessions. Those desiring hybrid or synchronous sessions were mostly parents. They expressed that they perceived these format styles as providing more accountability for program attendance and engagement. In contrast, youth primarily preferred asynchronous sessions, stating that it can feel uncomfortable to discuss health-related topics in front of other participants. The recommended length of intervention sessions was 40 to 60 minutes. When asked about factors that make it challenging to attend intervention sessions, most families felt that schedule conflicts and other household responsibilities (eg, cleaning and cooking) could be potential barriers. Suggestions for making intervention sessions engaging included having activities (eg, worksheets, meal planning, and games) and making the content interactive with quizzes and fun facts. The original intervention included a tasting experience where families were provided with a small, healthy snack to taste, with the purpose of exposing them to potentially new fruits or vegetables or new recipe ideas. When asked about the feasibility of conducting the tasting experience on their own at home, almost all participants (34/36, 94.4%) expressed that they preferred to conduct the tasting experience at home. Perceived barriers included not having all the ingredients or cooking equipment needed, the need for simple instructions, and having a busy schedule with little time for complicated recipes or additional grocery shopping. Facilitators of conducting the tasting experience on their own included having a planned schedule of recipes at the beginning of the intervention, so that they could be prepared, having the entire family participate and help prepare the food items, providing a gift card for the food items needed, and having instructional videos to demonstrate the recipes. With regard to their health education needs, families shared that it would be important to be able to ask questions about the content. They also expressed the desire to receive suggestions for healthy meals and snacks. When asked about the best digital format for asking questions, 52.7% (19/36) of participants preferred a discussion board, and 33.3% (12/36) recommended using SMS text messaging to send questions to the research team, with few respondents selecting email or social media. To further evaluate accessibility, we asked families if they would need access to the internet or digital devices to participate. While 83.1% (31/36) of participants reported that they have reliable internet connectivity and access to multiple internet-connected digital devices, 16.6% (6/36) were not sure. Further discussion revealed that families who were not sure felt that there might not be enough devices in the household for all family members to participate in the program.

### Step 2 Results

Given mixed findings regarding the session format, research and community experts recommended asynchronous intervention sessions as well as monthly, live synchronous sessions. The purpose of having asynchronous sessions would be to add flexibility, given that schedule conflicts are a common barrier to participation. It was recommended that synchronous sessions should focus solely on providing a platform for asking questions about the nutrition education content as well as fostering discussion around successes and challenges with progress toward behavioral goals to meet needs for engagement and accountability. This may also foster social support and relatedness between families. On the basis of the experience of the expert panel, it was suggested that live, synchronous sessions be conducted separately for parents and youth. This would make it more comfortable for youth and parents to ask questions and discuss sensitive topics. When discussing feedback on making the intervention engaging, community experts shared the importance of setting the tone for engagement by hosting an orientation session where families are informed of expectations, given clear instructions on how to complete intervention activities, and provided a call to action, encouraging the whole family to commit to supporting and working together on this journey. Other strategies that have been successful in increasing engagement include small incentives for completing intervention activities such as raffle prizes, movie tickets, and small exercise resources (eg, jump ropes, yoga mat, and resistance bands). With regard to adapting the food tasting experience, community experts had previous experience using gift cards for the food experience and found that few participants followed through with making the recipe on their own. Other members of the expert panel shared past experiences using a food box given to families at the beginning of the intervention. Panel members recommended that we test an adapted version of the food experience to gather more information on the feasibility of families completing this activity on their own. It was also suggested that we develop a space on the digital platform to provide additional recipes to meet the need for healthy meal and snack ideas. Both panels advised that we should be responsive to the need for a discussion board to provide an additional platform for asking questions. In light of participant concerns that they may not have enough internet-connected devices, panel members also advised that our platform should be adaptable so that it can be easily viewed on multiple types of devices.

### Step 3 Results

The sample intervention session included an adapted version of the food tasting experience to be conducted at home, education content, and time for setting a SMART goal. The sample session lasted 28 minutes. To participate in the food tasting experience in their home, all families were given instructions on preparing ingredients for a healthy snack of apples and peanut butter. The recorded session started with a demonstration of the food preparation, followed by a series of questions that encouraged mindful eating (1.5 min). The nutrition education content consisted of PowerPoint slides with one instructor delivering content (26.5 min). Slides were designed at a fourth- to sixth-grade reading level and included images and brief explanations, and participants were provided with an accompanying sample workbook that provided additional explanation of the education content and a SMART goal worksheet. Findings from the evaluation survey following the viewing of the sample session are presented in Table 2.

**Table 2.** Community feedback on sample educational session (n=21).

Theme	Values, n (%)
Food experience	
Perceptions of food experience	
Liked the food experience	18 (81)
Neither liked nor disliked the food experience	1 (5)
Did not like the food experience	0 (0)
Did not participate	3 (14)
Willingness to participate in food experience	
Would be willing to make the food recipe every week	21 (100)
Would NOT be willing to make the food recipe every week	0 (0)
Barriers to food experience	
Difficulty getting ingredients	8 (38)
Time or schedule constraints	6 (29)
Nothing	7 (33)
Facilitators of food experience	
Deliver a weekly food box	8 (38)
Grocery store gift card	8 (38)
Pickup order at grocery store	5 (24)
Educational content	
Most liked session components	
Everything	4 (19)
Concepts or learning objectives	11 (52)
Visual aids or slides	4 (19)
Suggestions for improvement	
More engaging speakers	2 (10)
Shorter session	3 (14)
Perceptions of session length	
Too long	2 (10)
Just right	17 (81)
Too short	0 (0)
Perceptions of engagement	
The *overall session* was engaging	18 (86)
The *teachers* were engaging	17 (81)
The *activities* were engaging	17 (81)
Self-reported comprehension	
The session was easy to understand	18 (86)
The learning objectives were clear	21 (100)
The illustrations and examples were easy to comprehend	19 (90)
Perceptions of feasibility	
Very likely to participate in the full 12-wk program	16 (76)
Not sure if I would participate in the program	5 (24)
Not likely I would participate in the full 12-wk program	0 (0)

Most participating families, except for 3, made the snack on their own (n=18/21; 85.7%); however, all participants reported that they were willing to participate in the tasting experience on their own. Once again, audio from focus groups was transcribed and coded using a thematic content analysis approach to identify emergent themes regarding feedback on the live session. The focus group discussion revisited potential challenges, which continued to include obtaining the ingredients and time constraints. However, some participants expressed that completing the tasting experience on their own would not be challenging. When presented with a list of cooking equipment, most families reported having access to the most common appliances and utensils, including measuring cups, a stove, a blender, a microwave, and cooking utensils, suggesting that access to cooking equipment was less likely to be a barrier. Recommended resources for making the tasting experience more feasible included a weekly box of food items, a gift card, and curbside pickup orders at the grocery store. Participating families reported that the content was easy to comprehend, the learning objectives were clear, and there was high agreement that the examples used to illustrate concepts were easy to understand. While some felt the session was too long, most felt the length was just right. Additionally, most participants felt that the content was engaging and that the health educator delivering the content was also mostly engaging. This feedback was used to adapt the sessions in the following ways: (1) session format was maintained as asynchronous, virtual; (2) families were provided with a food box containing nonperishable items (eg, peanut butter, tuna, and oatmeal), and the food tasting experience was limited to 4 sessions designed to be quick and made with affordable ingredients; (3) instructional delivery of the content was changed from 1 to 2 instructors who used conversational speech; (4) session length was kept at 30 minutes or shorter; and (5) activities were kept as central parts of sessions with clear instructions and cues from instructors on when and how to complete activities.

### Step 4 Results

In collaboration with an online course platform, Learnworlds, we were able to design a website and accompanying mobile smartphone app to deliver the intervention. This platform is a password-protected website, and the app mirrors the website, allowing participants to seamlessly access content across multiple devices (eg, computer, tablet, and smartphone) and web browsers (eg, Google and Safari). This platform allows for content to be presented on demand but also allows for live sessions to be hosted by connecting a personal or institutional Zoom account to the eLearning platform. To address suggestions for engagement, Learnworlds facilitates the development of interactive session videos with features including pop-ups, animations, quiz questions, and subtitles to enhance comprehension. This platform is dual language, and participants can view the website as well as subtitles and content in English and Spanish. In response to feedback from participants, we designed additional pages within the website to host a discussion board, a digital e-workbook, healthy meal and snack recipes, and a closed-loop messaging system so that participants can ask the research team questions about the content or technical issues experienced. The discussion board has multiple channels and allows participants to post digital media. This will allow us to host a channel for discussing challenges and successes in achieving behavioral goals as well as other channels for posting activities, such as displaying recipes made or posting pictures of participants engaging in healthy behaviors, to foster engagement among participants. The digital workbook will mirror the physical workbook and provide an additional opportunity for participants to access program resources. In response to the need for healthy meal ideas, the research team developed a brief series of 4 food demonstration videos and used the recipes page to recommend meal and snack ideas that are healthy and culturally appropriate. To assess engagement, the Learnworlds dashboard provided the research team with metrics for each individual participant, including the number of logins, the amount of time spent on the platform per login, and the completion rate of each task (eg, minutes of a video session that were played).

### Step 5 Results

Observations from trained research team members during the “think-aloud” assessment demonstrated that participants perceived the online learning platform to have moderate to high usability. Two parents failed to complete all 6 tasks within the time limit; however, they completed 4 of the 6 tasks (15 min). In contrast, youth participants completed all 6 assessment tasks within the time limit. Thematic content analysis in NVivo was used to identify common themes expressed by parents during the follow-up focus group and included difficulty in finding the login button (2/4, 50%, parents), finding course content (3/4, 75%, parents), and confusion about whether to fill out SMART goals in the digital workbook or in their paper workbook (2/4, 50% parents). Feedback obtained regarding the design of the platform mostly came from parents and included recommendations for increased font size, larger icons for sessions, more contrasting colors, larger tabs for additional pages, such as the discussion board, and creating multiple pathways for accessing the additional pages. Regarding functionality, participants felt that the digital workbook, which was uploaded in PDF form, should be more interactive and “fillable.” All other aspects of the platform functioned as designed.

### Step 6 Results

After careful review of the results from the think-aloud assessment, expert panel members recommended that we develop tutorial videos that should be located on the landing page so that participants have a reference tool for logging in, navigating to session content, and asking questions. It was also suggested that a brief training be given at orientation before the intervention. In this meeting, we discussed any remaining items that had yet to be finalized, such as the tasting experience. The experts recommended that food tastings should be implemented monthly and that a food box with nonperishable food items should be given to families at the beginning of the intervention (eg, canned garbanzo beans for hummus, peanut butter, and tuna) to reduce financial and time burdens. The expert panel proposed that families should be awarded extra “points” for making additional recipes. Given that the discussion board allows for photos and the use of hashtags, it was suggested that we create a discussion board channel dedicated to sharing experiences in making and trying healthy recipes so that families can encourage each other toward healthy eating.

### Step 7 Results

The feasibility pilot study to test the feasibility and acceptability of the digital diabetes prevention program began in October 2024. Feasibility criteria that were evaluated within the pilot include the following: (1) recruit 40 Hispanic adolescents aged 14 to 16 years; (2) retain 80% of participants for postassessments; (3) integrity of the study protocol defined as ≥70% completion of content sessions, Fitbit wear on ≥5 d/wk (Fitbit LLC) with response to 80% of text messages when prompted; (4) ≤10% technical issues; and (5) obtain ≥80% satisfaction from participants. After completion, we will continue to incorporate feedback from participants to iteratively refine this prevention program.

## Discussion

### Principal Findings

The COVID-19 pandemic accelerated the growth and implementation of telemedicine and the remote, digital delivery of behavioral health services. The Centers for Medicare & Medicaid Services temporarily changed federal and state health policies to expand access to telemedicine for all Medicare beneficiaries. These policy changes were extended through 2024, and it is believed that they may be extended in the future [20]. These types of policy changes could pave the way for disease prevention programs to be remotely delivered via digital platforms, making them more accessible and underscoring the need for evidence-based DHIs. However, there is a significant gap in the literature regarding frameworks and guidelines for adapting interventions to a digital format [21]. This study describes the process by which an existing, evidence-based diabetes prevention program for Hispanic youth and families was adapted for remote, digital delivery, using a co-design approach. Few studies have used similar qualitative and engagement strategies to co-design DHIs among adolescents, specifically Hispanic adolescents [22,23]. Use of the co-design approach provided novel insights and an in-depth description of the perceived needs, preferences, and contextual factors that impact participation in a digital diabetes prevention program among Hispanic youth and families.

The use of a co-design approach led to increased accessibility across several dimensions, including access to technology, usability of the platform, access to the information, which speaks to the user’s ability to understand and comprehend health-related information in their language of preference, and the format and implementation of the program. This is consistent with a recent scoping review on DHIs for youth that reported high levels of acceptability and accessibility among studies that use collaborative development methods [22]. This program is accessible across multiple devices, many of which are already owned and operated by Hispanic families, to address concerns regarding limited devices. Selecting a dual-language platform and engaging end users in the evaluation of the platform and content worked to ensure that the platform is easy to use and that nutrition information is easy to comprehend [11]. A review of barriers and facilitators to accessibility in DHIs found that having digital tools available in the participant’s first language is critical to increasing participation and engagement with intervention content [24]. The asynchronous format was selected to meet requests for flexibility to overcome time and schedule conflicts. Schedule conflicts and lack of program flexibility are consistently named among the most common reasons parents withdraw their children from traditional disease prevention interventions, making it important to provide a delivery format that meets the needs of our focus population [25]. It was of high importance, particularly among parents, to have the ability to ask questions, and several mechanisms were developed to provide this support, including live, synchronous sessions with instructors, a discussion board, and a closed-loop messaging system. Human support is a core component across most behavior change interventions, and previous studies have found that it is critical to retain modifications that still allow for guidance, support, and feedback after adaptations for digital delivery have been made [26].

In addition to accessibility, significant consideration was given to making sure that sessions were engaging [24]. This is consistent with other studies that have highlighted the importance of having engaging content, as digital strategies with low visual appeal or a lack of interactivity are seen as frustrating and boring [24]. Several interactive features were integrated into session videos (eg, quizzes), and the use of other components, such as the discussion board, will also encourage engagement between participants. These design elements, particularly games, challenges, and social elements, have been shown to enhance engagement [24]. It was also critical to the team to be able to assess engagement. With a user dashboard through the Learnworlds platform, all participant activity can be monitored in great detail, including the length of time participants engaged in a session video and the completion percentage of all activities. These types of engagement metrics will greatly contribute to understanding the feasibility and acceptability of using a digital learning platform for disease prevention. Additionally, engagement is tied to effectiveness, and therefore, more rigorous measures of participant engagement with digital tools can be used to understand efficacy within DHIs [27].

It is important to recognize that youth and parents reported different needs, preferences, and digital literacy skills. For example, some parents were more open to synchronous sessions, whereas all youth preferred asynchronous sessions. While all youth were able to easily complete tasks associated with the think-aloud assessment, parents found the tasks to be more challenging. Differences across age in needs and preferences for DHIs have been previously reported and are attributed to the fact that today’s youth have an increased level of comfort with technology due to high exposure to technology from an early age, and they also exhibit an increased willingness to receive health benefits from digital health tools [28]. Low digital health literacy among adults, including the skills and knowledge needed to navigate digital tools, is a significant driver of poor engagement in DHIs, further warranting the use of co-design approaches to develop a feasible platform [29]. At times, it was challenging to balance the needs and preferences of youth and parents in the context of a family-based intervention, which is in line with best practices for disease prevention [30]. This led to decisions such as hosting live sessions separately for youth and parents, which may provide the opportunity to address separate needs.

Our study also demonstrates that some components within in-person interventions are more challenging than others to adapt for digital delivery, such as the food tasting experience. In the original intervention, this component was used to teach basic principles of hunger and fullness cues and mindful eating and to expose participants to new fruits and vegetables to encourage greater intake of healthy foods. This was a very acceptable and engaging component within the original intervention; however, adapting this component proved to be challenging. The new delivery context, such as a digital platform, can pose new challenges during the adaptation process, leading to the need to make adjustments based on the user’s needs and preferences. Previous studies have reported that challenging adaptations within DHIs must be iteratively developed and tested to ensure that the “active ingredients” of the original intervention are retained and remain effective within the new digital context [31]. In this study, the use of the co-design process allowed us to work iteratively through a cycle of feedback between experts and community members until the most appropriate solution was identified.

### Limitations

This study is not without limitations. Given the focus on high-risk Hispanic adolescents and families, our findings may have limited generalizability to other age groups or ethnic and racial subgroups. However, this framework can be used to adapt and co-design DHIs to meet the needs and preferences of other populations. Additionally, more research is needed to confirm that adapting DHIs using a co-design framework can improve the feasibility, acceptability, and efficacy of digital strategies. As this study progresses toward a feasibility pilot trial, the adaptations and decisions made through this co-design process will receive further testing and feedback from Hispanic youth and families, which will lead to additional refinements and provide evidence on the importance of engaging youth through co-design processes.

### Conclusions

This study provides a framework for adapting an evidence-based diabetes prevention program for remote delivery via a digital platform. The use of a co-design approach served to increase the accessibility and engagement of the adapted digital intervention by informing the format, the design of the platform, and the design features. In addition to identifying the preferences and needs of the focus population for a digital disease prevention program, this study also highlighted challenges in adapting in-person interventions to a digital format. Certain components that were integral to the in-person intervention, such as the tasting experience, required greater input from end users and experts to adapt for remote delivery. This adaptation process was followed by a 12-week feasibility pilot to further assess the feasibility and acceptability of the digital diabetes prevention intervention. Describing the adaptation process in collaboration with end users will inform future studies aiming to adapt evidence-based interventions to a digital platform.

## Supplementary material

10.2196/67800 Multimedia Appendix 1 English sample session survey.

## Abbreviations

DHI: digital health intervention
DPP: Diabetes Prevention Program
SMART: specific, measurable, achievable, relevant, and time-bound
T2D: type 2 diabetes
