# Supplementary material for: Community Participatory Co-Design and Development of a Digital Diabetes Prevention Education Program for Hispanic Families With Obesity: Mixed Methods Study
Source: JMIR Form Res. 2026 Feb 11;10:e67800. doi: 10.2196/67800 (PMC12893523; doi:10.2196/67800)
Supplement: Multimedia Appendix 1 [file formative-v10-e67800-s001.docx]

**Appendix A:**

English Sample Session Survey

Please watch the video below as if you were participating in a weekly wellness class and fill out the below handout as you watch. Then, answer the questions below.

What is your ID number?

Are you a parent or teen?

1. Parent
2. Teen

How was the video quality and clarity?

A. High quality- I could see everything in enough detail

B. Medium quality- I could see most things but some were blurry

C. Low quality- I thought most of it was blurry

How was the audio quality and clarity?

A. High quality- I could hear everything clearly and follow along

B. Medium quality- I could hear most things clearly but some parts were fuzzy

C. Low quality- I thought most of it was hard to hear clearly

If you were to participate in Fit24+ for 12 weeks, how would you prefer to watch the nutrition education videos?

1. I would prefer to watch pre-recorded videos with my family whenever we are available.
2. I would prefer to attend live nutrition education sessions online with other families who are also in the program at a set time every week.
3. I would prefer to attend some sessions online with other families and to have some of the sessions pre-recorded

-What, if anything, would make it difficult to get your family together for online nutrition education sessions once a week for 12 weeks?

  [Text box response]

Did you watch the whole video?

1. Yes
2. No

What is the best way to have a conversation about weight with someone?

1. Focus on their appearance and how much they weigh
2. Focus on health behaviors, starting with open-ended questions
3. Tell them to go on a diet
4. Compare the way they look to other people

In the video, what symbol was used for LDL cholesterol?

1. A bulldozer
2. A truck
3. A construction worker
4. A construction barrier

What did you think about making and trying the snack with your family?

1. I liked making and trying the snack with my family
2. I did not like making and trying the snack with my family
3. I’m not sure….or I neither liked or disliked it
4. I did not actually make the snack while watching the video

How likely is it that you would sign up to participate in this 12-week program?

1. It is very likely that I would participate in this program.
2. It is not very likely that I would participate in this program.
3. I’m not sure if I would or would not participate in this program.

IF NOT VERY LIKELY: Can you tell us more about why you would not participate

in a program like this?

What was your opinion on the length of the nutrition education session?

1. I thought the session was too long.
2. I thought the session was too short.
3. I thought the session length was just right.

Please select how much you agree or disagree with the statements below.

1. Overall, the nutrition education session was engaging….
2. The teachers in the nutrition education session were engaging…
3. The activities in the nutrition education session were engaging…
4. The information in the nutrition education session was easy to understand…
5. The learning objectives in the nutrition education session were very clear…
6. The examples used in the nutrition education session were easy to follow….
7. I liked the visual aids used in the nutrition education session…
8. I liked using the workbook to answer questions and write out notes…
9. I would like to receive additional information and helpful tips via text message
10. I would like to receive additional information and helpful tips via social media
11. What else here??? We need to understand their perceptions of the nutrition education session

“If you selected anything other than agree or strongly agree, can you please tell us more? For example, if you selected “strongly disagree” for the first statement, could you describe why you thought the session was not engaging?”

[Text box response]

What would make the session easier to understand? (multiple checkboxes)

1. More explanation of Bloodstream Highway
2. Less explanation of Bloodstream Highway
3. More explanation on weight and what it means for health
4. Less explanation on weight and what it means for health
5. More explanation on what to say to the doctor
6. Less explanation on what to say to the doctor
7. Other [free response box]

What would make the session more engaging/ interesting?

1. More pictures
2. Fewer pictures
3. More words on the slide
4. Fewer words on the slide
5. More handouts
6. Fewer handouts
7. None of the above: I already found it fully engaging.
8. Other: free text

If we were to create a platform for asking questions, what platform would be the easiest for you to use?

1. I would like to post my questions on a website discussion board
2. I would like to be able to post my questions on Social media
3. I would like to text my questions to the instructor
4. I would like to email my questions to the instructor

Would you be willing to make a snack with your family every week during the 12-week program?

A. Yes, I would be willing to make a snack with my family every week during the 12-week program.

B. No, I would not be willing to make a snack with my family every week during the 12-week program.

IF NO: Can you tell us more about why you would not be willing to make a snack with your family every week?

A: I’m just not interested

B: I’m worried about buying the ingredients or going to the store to get the ingredients

C: My work schedule would not allow for this

D: My family doesn’t have time to do this every week.

E:  Other [free response box]

What, if anything, would make it difficult to make a snack every week with your family for 12 weeks?

[Text box response]

What could we provide to help make it easier to make a snack every week with your family during the program?

1. Send each family a box of the ingredients to make the weekly snacks
2. Send each family a gift card so that they can go to the grocery store and purchase the ingredients to make the weekly snacks
3. Grocery store pick-up
4. Other options we can offer

What cooking utensils do you have available at home to help you make the snacks or meals we demonstrate in the classes? Please check all that apply.

1. Microwave
2. Blender
3. Oven
4. Stove top
5. Other [free response box]

What did you like most about the class?

[Text box response]

What would you change about the class?

[Text box response]

Would you like to be contacted about our future research project involving FitBits and physical activity (it will be paid)?

1. Yes
2. No
3. Not sure

If we have any questions about any of your responses, can we call you over the phone to find out more information?

1. Yes
2. No
